# Area deprivation and attachment to a general practitioner through centralized waiting lists: a cross-sectional study in Quebec, Canada

**DOI:** 10.1186/s12939-018-0887-9

**Published:** 2018-12-04

**Authors:** Mélanie Ann Smithman, Astrid Brousselle, Nassera Touati, Antoine Boivin, Kareen Nour, Carl-Ardy Dubois, Christine Loignon, Djamal Berbiche, Mylaine Breton

**Affiliations:** 1 Centre de recherche Charles-Le Moyne - Saguenay Lac-St-Jean sur les innovations en santé, Université de Sherbrooke, Longueuil Campus, 150 Place Charles-Le Moyne, Suite 200, Longueuil, Quebec J4K 0A8 Canada; 2 School of Public Administration, University of Victoria, 3800 Finnerty Rd, Suite A302, Victoria, British Columbia V8P 5C2 Canada; 3 Centre de recherche sur la gouvernance, École nationale d’administration publique, 4750, Avenue Henri-Julien, Office 5117, Montreal, Quebec H2T 3E5 Canada; 4 Centre de recherche du Centre Hospitalier de l’Université de Montréal, Université de Montréal, 900 Rue Saint-Denis, Montreal, Quebec H2X 0A9 Canada; 5 Direction de santé publique, Centre intégré de santé et des services sociaux - Montérégie-Centre, 1255 rue Beauregard, Longueuil, Quebec J4K 2M3 Canada; 6 Faculty of Nursing, Université de Montréal, 2375, chemin de la Côte Ste-Catherine, Office 5103, Montreal, Quebec H3T 1A8 Canada

**Keywords:** Physicians, family, Physicians, primary care, Primary health care, Health services accessibility, Socioeconomic factors, Healthy equity, Waiting lists, Cross-sectional, Canada, Quebec

## Abstract

**Background:**

Access to primary healthcare is an important social determinant of health and having a regular general practitioner (GP) has been shown to improve access. In Canada, socio-economically disadvantaged patients are more likely to be unattached (i.e. not have a regular GP). In the province of Quebec, where over 30% of the population is unattached, centralized waiting lists were implemented to help patients find a GP. Our objectives were to examine the association between social and material deprivation and 1) likelihood of attachment, and 2) wait time for attachment to a GP through centralized waiting lists.

**Methods:**

A cross-sectional study was conducted in five local health networks in Quebec, Canada, using clinical administrative data of patients attached to a GP between June 2013 and May 2015 (*n* = 24, 958 patients) and patients remaining on the waiting list as of May 2015 (*n* = 49, 901), using clinical administrative data. Social and material area deprivation indexes were used as proxies for patients’ socio-economic status. Multiple regressions were carried out to assess the association between deprivation indexes and 1) likelihood of attachment to a GP and 2) wait time for attachment. Analyses controlled for sex, age, local health network and variables related to health needs.

**Results:**

Patients from materially medium, disadvantaged and very disadvantaged areas were underrepresented on the centralized waiting lists, while patients from socially disadvantaged and very disadvantaged areas were overrepresented. Patients from very materially advantaged and advantaged areas were less likely to be attached to a GP than patients from very disadvantaged areas. With the exception of patients from socially disadvantaged areas, all other categories of social deprivation were more likely to be attached to a GP compared to patients from very disadvantaged areas. We found a pro-rich gradient in wait time for attachment to a GP, with patients from more materially advantaged areas waiting less than those from disadvantaged areas.

**Conclusion:**

Our findings suggest that there are socio-economic inequities in attachment to a GP through centralized waiting lists. Policy makers should take these findings into consideration to adjust centralized waiting list processes to avoid further exacerbation of health inequities.

## Background

Access to primary healthcare (PHC) is an important social determinant of health and improving access to PHC is considered to be a key strategy to reducing health inequities [1]. Although improving equity has been a central aim of health reforms in many OECD countries, inequities in access to PHC persist [2, 3]. Even in countries with universal healthcare systems, like Canada, access has been found to vary, not only according to patients’ medical need, but also according to factors such as income, education, social support and region of residence [2, 4, 5]. Having a regular general practitioner (GP) has been shown to improve access to PHC [6, 7].

However, many studies have found inequities in having a regular GP [6, 8–11] and in the likelihood of having visited a GP [2, 5, 12], with patients with lower levels of income, education and social support having poorer access to a GP. Although in Canada GPs are key to accessing PHC as they are often the first point of contact to the healthcare system [13, 14], 15% of the population does not have a regular GP [14] - a large proportion compared to seven other OECD countries [15]. Quebec is the Canadian province with the highest proportion of patients reporting they do not have a regular GP [16], despite having a GP to population ratio higher than the Canadian average (116 vs. 111 GPs per 100,000 population) [17]. In Quebec, patients are formally attached to a GP, meaning that patients are officially registered on GPs’ patient panels through an agreement signed on a voluntary basis during the first visit [17]. Formal attachment is strongly encouraged by the Ministry of Health and Social Services and is intended to increase GPs’ accountability to their patients and to foster a continuous physician-patient relationship [18]. As of 2015, only 67.8% of the population was formally attached to a GP [19]. Over 65% of unattached patients report not being able to find a GP as the main reason they are unattached [10]. One of the main explanations for this large proportion of unattached patients is that GPs are required to dedicate a part of their time to regionally determined particular medical activities (*activités médicales particulières*) such as practicing in the emergency department or in long-term care facilities [17]. Consequently, GPs in Quebec have fewer hours available to care for attached patients [17].

In order to improve access to PHC, seven Canadian provinces (Ontario, Quebec, Manitoba, Nova Scotia, Prince-Edward-Island, British Columbia and New Brunswick) have implemented centralized waiting lists (CWLs) to help patients find a GP [20]. CWLs have a single intake point for patients’ demand for a given service, may prioritize patients based on certain criteria, and help link patients to a provider from within a pool of providers. CWLs have been implemented in many fields of healthcare, namely for elective surgeries, transplants, occupational therapy, mental health services and referrals to specialists [21–25]. CWLs, with clear and respected guidelines for managing patient and provider lists, have been suggested as way to equalize wait times for patients with similar medical needs and to increase fairness in access to health services [26–28]. In other words, because CWLs standardize processes to access a given service, they could have the potential to reduce socio-economic inequities in attachment to GPs.

However, to our knowledge, there have been no studies on socio-economic inequities of CWLs for attachment to a GP. Yet, this information is essential to understand whether these CWLs are improving equity in access to a GP. In this paper, we aimed to fill this gap by analyzing the association between socio-economic factors and patient attachment to a GP through CWLs in Quebec. More specifically, the objectives were to examine the association between social and material deprivation and 1) likelihood of attachment, and 2) wait time for attachment to a GP through CWLs. First, we provide information on Quebec’s CWLs and how they work; followed by contextual information on Quebec’s healthcare system and a description of our methods; subsequently, we present descriptive statistics and findings for both objectives in the results section; we then discuss the main findings of our study with regards to inequities in registration on CWLs and attachment to a GP, the main implications for policy and the limitation of the study. This paper is part of a larger study evaluating the implementation and effects of CWLs for unattached patients in Quebec [29].

### Centralized waiting lists for attachment to a GP

In Quebec, CWLs, the *guichets d’accès pour la clientèle orpheline*, were implemented in 2008 to increase the number of patients formally attached to GPs and prioritize attachment for patients with urgent health needs [30]. Over a million patients having been attached to a GP through these CWLs across the province [31]. Each CWL is staffed by a nurse, a clerk and a medical coordinator, a local GP, who manage the CWL for the local health network, according to general provincial guidelines.

CWLs are intended to help patients find a GP, as opposed to patients having to contact each clinic to see if a GP is available to attach them, but are not mandatory for GPs or patients. Unattached patients are not automatically registered on the CWLs. Those want to register on the CWLs can fill out an online or paper form or contact the CWL by phone. Health providers (e.g. physician at walk-in clinic or emergency department, social worker) may also fill out a CWL registration form on behalf of an unattached patient. Approximately 423,000 patients were waiting for attachment to a GP on a CWL in the province in 2015, which represent about 17% of all unattached patients in Quebec. Other patients may not know about CWLs, may have chosen not to register on CWLs because they are looking for a GP on their own or because they are not looking for a GP. A patient may be formally attached to a GP through the CWLs or upon finding a GP that is willing to attach them through other channels such as at a walk-in clinic or through relatives or friends who ask their GP on the patient’s behalf. GPs who are available to attach new patients may use the CWLs to find new patients or may choose to attach patients outside the CWLs (e.g. patients they meet at walk-in clinics or relatives of their current patients).

Upon registration, patients’ contact, demographic and medical information is collected. Patients are asked to self-report medical conditions on the registration form (may also be filled out by a provider or over the phone as aforementioned). The CWL nurse uses this information, and may contact the patient by phone to obtain additional information, to assign patients one of five clinical priority levels, from priority 1 (most urgent) to 5 (least urgent), according to the guidelines in the provincial reference framework and clinical judgment. The clinical priority levels, based on patients’ medical needs, were implemented to help prioritize attachment of patients with the most urgent needs and are linked to recommended wait times [30]. The wait times for each priority level are simply recommendations to help guide prioritization and are not enforced. Patient information is updated in the CWL database only if the patient contacts the CWL staff to notify the staff of any changes.

CWL patients are generally attached to a GP based on the availability of GPs in the area, clinical priority level, registration date and GPs’ preferences and scope of practice. GPs’ attachment of new patients through the CWLs is voluntary: GPs can contact the CWL intermittently to request the desired number of new patients and may specify their preferences for certain types of patients. The CWL nurses are responsible for selecting patients from the CWL for GPs who are willing to attach new patients and have some discretionary power in selecting patients for attachment, for instance to respect a GP’s scope of practice or preference of certain types of patients. GPs may return patients they have not been able to reach, who have missed their first appointment or who do not fit their scope of practice to the CWL. A recent report in Quebec found that in about 35% of cases where patients were sent back to the CWL without attachment to a GP it was because the GP felt they were medically or personally incompatible with the patient [32].

GPs receive a one-time financial incentive to attach patients from the CWL, with a larger incentive for patients with a least one of 19 medical vulnerability codes (e.g. cancer, mental health problems, intellectual disability) as defined by Quebec’s Health Insurance Board, the *Régie de l’Assurance Maladie du Québec* (RAMQ), or being 70 years old or older [33]. Between 2013 and 2015, financial incentives for GPs for attaching patients from CWLs were: 100$CAD for patients without a medical vulnerability code, 208$CAD for patients with at least one medical vulnerability code or 250$CAD for patients with a mental health or addiction problem [34]. GPs could receive financial incentives for attaching up to 150 new patients per year, with the exception of newly practicing GPs (4 years or less) who were not limited [34].

While CWLs for attachment to a GP have been implemented in most Canadian provinces, we have not found evidence of this type of CWL outside the Canadian context [20, 35]. Within Canada, a recent study comparing the design of CWLs for attachment to a GP across seven provinces found that they had similar registration processes, but varied in how patients were prioritized for attachment and in financial incentives for GPs [20]. The study also reported that CWLs across Canada faced similar challenges in attaching vulnerable and complex patients and in the limited capacity to meet the demand for attachment [20]. Previous studies on Quebec’s CWLs, using aggregated data have found that, despite larger financial incentives for attaching patients with health conditions, GPs showed a preference for attaching healthy patients [35] and that there are large variations in how CWLs perform across the province [30]. Moreover, although no research on CWL patients’ socio-economic characteristics has been done to date, the equity of attachment to a GP through these CWLs has been called into question by the Auditor General in Quebec [36].

## Methods

### Study setting

#### Context: Healthcare system in Quebec

In Canada, each province and territory is responsible for organizing the delivery of most healthcare services [37]. Quebec, the second most populous Canadian province (population: 8.3 million) [38], provides publically funded universal health insurance for medical services. The Quebec Ministry of Health and Social Services determines the provincial healthcare system’s priorities. The majority of GPs are paid on a fee-for-service basis. The large majority of primary care practices are managed by GPs who are self-employed, but paid by the government. Medical services provided by GPs are paid by the public provincial health directly. Approximately 20% of GPs are salaried and work in local community services centers which are managed by the government [35]. During the period under study, the province was divided into 94 geographically delimited local health networks which were created to address the different needs of the population in each area and implement a “population-based responsibility” in healthcare planning and delivery [39].

### Study design

We conducted a cross-sectional study of CWLs for attachment of patients to a GP using clinical administrative data from Quebec, Canada.

### Data source

We used administrative data from Quebec’s information system for CWLs for unattached patients: the *système d’information des guichets d’accès pour la clientele orpheline* (SIGACO) database. This database compiles information for every patient requesting a GP through the CWLs. The data available in the SIGACO database is limited to attachment to GPs through CWLs and does not contain information on attachment outside CWLs.

Data is entered manually in the SIGACO system by the CWL’s secretary or nurse in each local health network. We extracted individual patient data from five CWLs, which were selected to compare and contrast the implementation and performance of CWLs with varying results for indicators such as number of patients attached to a GP, number of patients on the waiting list and proportion of medically vulnerable patients attached to a GP [30], as part of the larger study on CWLs [29]. Four of the five CWLs were located in urban local health networks (A, B, C and E) and one in a semi-urban (D) local health network. In total, these local health networks represent about 12% of Quebec’s population.

Data was extracted for two groups of patients: those attached to a GP through one of the five CWLs between June 1st, 2013 and May 31st, 2015 (*n* = 24, 958), and those who remained on one of the five CWLs (i.e. still waiting for attachment) as of May 31st, 2015 (*n* = 49, 901). This period was selected because it represented the most recent data available at the time of the study and because changes in financial incentives and CWL guidelines had major impact on pre-2013 attachment [35].

### Variables

#### Dependent variables

Our first dependent variable was likelihood of attachment to a GP through CWLs versus remaining on the waiting list, categorized as follows: 1) attached to a GP (*n* = 24, 958) and 2) waiting for attachment (*n* = 49, 901). Our second dependent variable was wait time for attachment to a GP through CWLs. Only patients attached to a GP during the period under study (June 2013 to May 2015) were included in this analysis (*n* = 24, 958), because their total wait time could be calculated, whereas total wait time for attachment for patients remaining on the waiting list was unknown. Wait time was defined as the number of days between the date of registration on the CWL, which could precede the study period, and date of attachment to a GP.

#### Main independent variables: Social and material deprivation

Our two main independent variables are social and material deprivation. Health services administrative databases created by provincial authorities generally do not contain socio-economic data on individual patients. To make up for this shortcoming, geographic proxies, socio-economic data from a census or large health survey linked to small geographic areas, are commonly used to study inequities [11, 40–49]. In Quebec, a social and material deprivation index was developed in the late 1990s by Pampalon and collaborators to overcome the absence of socio-economic information in administrative databases and was intended for use in research and health planning [11, 41, 49, 50]. Deprivation is conceptualized as “a state of observable and demonstrable disadvantage relative to local community or the wider society or nation to which the individual, family or group belongs” [49, 51]. The deprivation index is based on data from the Canadian census and relates to dissemination areas with populations of 400 to 700 individuals [49]. It includes socio-economic indicators grouped along two dimensions – material and social. Material deprivation is based on average personal income, proportion of persons without a high school diploma and employment ratio. Social deprivation is calculated using the proportion of persons living alone, the proportion of individuals who are divorced, widowed or separated and the proportion of single-parent families. Although the area-based deprivation index has been shown to underestimate inequities when compared to individual-level data, it produces statistically reliable estimates consistent with individual indicators and detects sizeable inequities between groups [52]. This index has been widely used to examine inequities in health and healthcare in Quebec and other Canadian provinces [41, 53–57]. For our analyses, we used the 2006 version of the deprivation index and linked it to patients’ postal codes in the SIGACO database. While a 2011 version of the index exists, it was calculated using the voluntary Canadian National Household Survey that replaced the census in 2011 and had a very high non-response rate which may have led to non-response bias and inaccurate results [58].

#### Control variables

We included sex and age as demographic variables. Based on previous work showing large variations in performance of CWLs between local health networks and no association between CWLs’ performance and proportion of the population attached to a GP [30], we controlled for local health network (A to E) but not for proportion of population attached to a GP per local health network. To control for health needs, we included: medical vulnerability (i.e. the presence or absence of at least one of 19 medical vulnerability codes or being older of 70 year old); clinical priority level (Priority 1 – most urgent, to Priority 5 – least urgent); and the total number of medical vulnerability codes as a proxy for complexity. In addition, we included the presence/absence of mental health problems (excluding major depression), major depression, addiction, intellectual disability, diabetes, COPD and active cancer. These conditions were identified as particularly important to control for because of their influence on likelihood and wait time for attachment in preliminary analyses and/or because they were identified as particularly influential in interviews with key stakeholders (GPs, CWL staff, policy makers) (unpublished results). For instance, stakeholders said that patients with active cancer were generally very rapidly attached, while patients with mental health or addictions problems waited much longer or remained on the waiting list because it was difficult to find GPs who would attach them (unpublished results). For the likelihood of attachment, we also controlled for wait time on the CWL: the number of days between registration on the CWL and May 31st, 2015 for patients who remained on the CWL and the number of days between registration and attachment for patients who had been attached to a GP. For the analysis on wait time for attachment, we were able to identify GPs to whom patients had been attached by their unique identifier in the database (*n* = 469 physicians) and, therefore, to account for their potential influence in the statistical model (see below).

### Analysis

We found less than 5% missing data and the listwise deletion method was employed for incomplete records. Univariate analyses (percentages, means, and standard errors) were used to describe the population under study. In order to assess the association between each independent and dependent variables, bivariate analyses were conducted (chi-square, t-test). To examine the association between social and material deprivation and the likelihood of attachment to a GP through CWLs, we used a multiple logistic regression, controlling for demographic, local health network, medical need and wait time variables, using forced entry, given that our selection of variables had a strong theoretical reasoning based on previous work (see Eq. 1). Odds ratios with 95% confidence intervals were determined for all variables.

Equation 1 Multiple logistic regression of likelihood of attachment to a GP through CWLs

1 $$ {\displaystyle \begin{array}{l}\mathrm{Log}\ \left[\frac{\mathrm{P}}{1-\mathrm{P}}\right]={\beta}_0+{\beta_1}^{\ast }{X}_{\mathrm{social}\ \mathrm{deprivation}}+{\beta_2}^{\ast }{X}_{\mathrm{material}\ \mathrm{deprivation}}+{\beta_3}^{\ast }\ {X}_{\mathrm{sex}}+{\beta}_4\\ {}{}^{\ast }{X}_{\mathrm{age}}+{\beta_5}^{\ast }{X}_{\mathrm{local}\ \mathrm{health}\ \mathrm{network}}+{\beta_6}^{\ast }{X}_{\mathrm{medical}\ \mathrm{vulnerability}}+{\beta}_7\\ {}{}^{\ast }{X}_{\mathrm{clinical}\ \mathrm{priority}}+{\beta_8}^{\ast }{X}_{\mathrm{number}\ \mathrm{of}\ \mathrm{health}\ \mathrm{conditions}}+{\beta_9}^{\ast }{X}_{\mathrm{health}\ \mathrm{conditions}}+{\beta}_{10}\\ {}{}^{\ast }{X}_{wait\ time}+\varepsilon \end{array}} $$

P: Probability of being attached; β_k_: beta coefficient for each variable; X_k_: variables included in the logistic regression model; ε: error.

For wait time for attachment to a GP through CWLs, the intraclass correlation related to GPs to whom patients had been attached was of 18%, indicating that a substantial proportion of variance in wait times was due to the GP. Because the intraclass correlation was larger than 5% [59], we used a multilevel model for examine the association between deprivation and wait time for attachment to a GP. We included GPs’ unique identifier at the higher level of our model, but no other variables related to GPs were included. We used a generalized linear mixed model, controlling for demographic, local health network and medical need variables (see Eq. 2).

Equation 2 Multilevel linear regression of wait time for attachment to a GP through CWLs

2 $$ {\displaystyle \begin{array}{l}\mathrm{Y}={\beta}_{00}+{\beta_{10}}^{\ast }{X}_{\mathrm{social}\ \mathrm{deprivation}}+{\beta_{20}}^{\ast }{X}_{\mathrm{material}\ \mathrm{deprivation}}+{\beta_{30}}^{\ast }\ {X}_{\mathrm{sex}}+{\beta_{40}}^{\ast }{X}_{\mathrm{age}}\\ {}+{\beta_{50}}^{\ast }{X}_{\mathrm{local}\ \mathrm{health}\ \mathrm{network}}+{\beta_{60}}^{\ast }{X}_{\mathrm{medical}\ \mathrm{vulnerability}}+{\beta_{70}}^{\ast }{X}_{\mathrm{clinical}\ \mathrm{priority}}\\ {}+{\beta_{80}}^{\ast }{X}_{\mathrm{number}\ \mathrm{of}\ \mathrm{health}\ \mathrm{conditions}}+{\beta_{90}}^{\ast }{X}_{\mathrm{health}\ \mathrm{conditions}}+{\mu}_{0j}+{\varepsilon}_{ij}\end{array}} $$

Y: Wait time for attachment; β_k_: beta coefficient for each variable; X_k_: variables included in the linear mixed model; ε: error.

For both analyses, we conducted sensitivity analysis with interaction terms between social and material deprivation, and between both deprivation indexes and all the control variables. The interaction terms were non-significant and had little influence of the estimates in the statistical models and were, therefore, excluded from the final analyses. All variance inflation factors (VIF) were inferior to 5 suggesting that there was no issues with multicollinearity in the logistic regression model. We used SAS 9.3 for all statistical analyses.

## Results

A total of 24, 958 patients were attached to 469 GPs between June 1st, 2013 and May 31st, 2015, while 49, 901 patients remained on the five CWLs as of May 31st, 2015. Average wait time (standard deviation-s.d.) for patients attached to a GP was 284 days (s.d. 350) and 458 days (s.d. 359) for patients waiting for attachment (*p* < 0.0001).

Table 1 compares patient characteristics in the two groups. Patients waiting for attachment differed significantly from patients attached to a GP. With regards to material and social deprivation, per definition, each deprivation quintile represents 20% of Quebec’s population. Compared to the distribution of deprivation indexes at the provincial level, we found that patients from the most materially advantaged areas (richest) represented 30.6% of patients on the waiting lists while patients from the most materially disadvantaged areas (poorest) represented a mere 11.65%, with a pro-rich gradient in proportions of patients on the waiting lists. This difference seems to be lessened among patients attached to a GP (23.27% most advantaged vs. 18.88% most disadvantaged). Conversely, we found that patients from very socially advantaged areas were slightly underrepresented (18.82%) and patients from very socially disadvantaged areas were overrepresented on the waiting list (29.49%) and with an even larger difference among patients attached to a GP (12.13% very advantaged vs. 37.78% very disadvantaged).

**Table 1:** Descriptive statistics for population under study (patients attached to a GP through centralized waiting list (*n* = 24, 958); patients waiting on centralized waiting lists (*n* = 49, 901), June 2013 to May 2015)

Descriptive statistics		All CWL patients (waiting for attachment & attached) (%)	Patients waiting for attachment (%)	Patients attached to a GP (%)	P (Chi-2 or t-test)
Material deprivation^a^	Very advantaged	28.16	30.60	23.27	< 0.0001
	Advantaged	21.96	23.16	19.56	
	Medium	17.37	17.05	18.01	
	Disadvantaged	18.46	17.55	20.28	
	Very disadvantaged	14.06	11.65	18.88	
Social deprivation^b^	Very advantaged	16.59	18.82	12.13	< 0.0001
	Advantaged	13.12	14.03	11.30	
	Medium	15.28	16.08	13.67	
	Disadvantaged	22.76	21.57	25.12	
	Very disadvantaged	32.25	29.49	37.78	
Sex (%)	Female	53.58	54.07	52.60	0.0001
Mean age (s.d.)^c^		42.13 (22.11)	42.56 (19.03)	41.29 (27.24)	< 0.0001
Local health network (%)	A	18.01	23.03	8.23	< 0.0001
	B	12.37	10.58	15.95	
	C	24.30	31.16	10.59	
	D	28.64	26.56	32.80	
	E	16.59	8.67	32.42	
Medical vulnerability (%)^d^	Vulnerable	31.38	23.95	46.23	< 0.0001
	Non-vulnerable	68.62	76.05	53.77	
Clinical priority (%)^e^	1 (most urgent)	1.95	0.37	4.62	< 0.0001
	2	15.86	6.33	31.90	
	3	12.57	8.63	19.22	
	4	25.54	27.06	22.98	
	5 (least urgent)	44.08	57.61	21.29	
Number of health conditions (%)^f^	None	72.48	79.20	59.04	< 0.0001
	1 condition	18.28	13.56	27.71	
	≥2 conditions	9.24	7.24	13.25	
Health conditions (%)	Mental health^g^	6.23	5.39	7.89	< 0.0001
	Major depression	1.91	1.47	2.80	< 0.0001
	Addiction	0.81	0.75	0.91	0.02
	Active cancer	3.90	2.29	7.11	< 0.0001
	Intellectual disability	0.48	0.40	0.62	< 0.0001
	Diabetes	5.35	3.52	9.02	< 0.0001
	COPD	3.86	3.19	5.22	< 0.0001
Mean wait time (s.d.)^c,h^		–	458 (359)	284 (350)	< 0.0001

^a^Area-based material deprivation

^b^Area-based social deprivation

^c^s.d.: standard deviation

^d^Medical vulnerability is determined by the presence of at least one of 19 medical vulnerability codes, as pre-determined by the RAMQ, or being older of 70 year old

^e^Clinical priority level as assigned by the CWLs’ nurses (ranging from Priority 1 – most urgent, to Priority 5 – least urgent)

^f^Number of medical vulnerability codes present

^g^Mental health problems excluding major depression

^h^Mean wait time in days: For patients waiting for attachment – number of days between CWL registration and May 31st, 2015; For patients attached to a GP – number of days between CWL registration and attachment. Not provided for all patients because measured differently for each group

A larger proportion of patients waiting for attachment were female (54%). Patients waiting for attachment were slightly older (1.27 years, *p* < 0.0001). The distribution of the two groups across local health networks was also significantly different. For instance, patients from LHN A made up 23.03% of patients waiting for attachment and only 8.23% of patients attached to a GP, while those from LHN E represented 8.67% of patients waiting for attachment and 32.42% of those attached to a GP. A much larger proportion of patients attached to a GP were medically vulnerable, had been identified as clinical priority 1, 2 or 3 and had one or more health conditions. The prevalence of mental health problems, major depression, addiction, intellectual disability, active cancer, diabetes and COPD was higher in patients attached to a GP.

Table 2 shows the distribution of patients across material and social deprivation quintiles, with regards to the other variables under study. A larger proportion of CWL patients from both materially and socially disadvantaged areas had been attached to a GP, compared to more advantaged areas. We observed a small gradient in mean age, with patients from socially and materially very disadvantaged areas being slightly younger. Patients from each deprivation quintile were unequally distributed across Local Health Networks, namely with Local Health Network E accounting for a large proportion of both socially and materially very disadvantaged patients. A smaller proportion of very socially and materially disadvantaged patients were prioritized as least urgent. A small gradient in the mean number of health conditions was observed, with patients from socially and materially disadvantaged areas having more health conditions. A slightly larger proportion of patients from both socially and materially very disadvantaged areas had mental health and addiction problems. In addition, a larger proportion of patients from materially very disadvantaged areas had an intellectual disability, diabetes and COPD.

**Table 2:** Distribution of material and social deprivation indexes according to the other variables used in this study (*n* = 74, 859; June 2013 to May 2015)

		Material deprivation^a^					Social deprivation^b^				
		very advant.	advant.	med.	disad.	very disad.	very advant.	advant.	med.	disad.	very disad.
Total sample (%)		28.16	21.96	17.37	18.46	14.06	16.59	13.12	15.28	22.76	32.25
Attachment though CWLs (%)	Attached to a GP	27.55	29.70	34.57	36.63	44.78	24.37	28.72	29.83	36.80	39.06
	Waiting for attachment	72.45	70.30	65.43	63.37	55.22	75.63	71.28	70.17	63.20	60.94
Sex (%)	Female	54.48	53.90	53.71	52.59	52.44	51.55	52.06	54.67	54.11	54.36
	Male	45.52	46.10	46.29	47.41	47.56	48.45	47.94	45.33	45.89	45.64
Mean age (s.d.)^c^		44.08 (21.90)	42.80 (22.14)	42.49 (22.15)	40.56 (21.94)	38.83 (22.22)	43.93 (21.89)	43.0 (22.79)	42.81 (22.49)	41.74 (22.29)	40.81 (21.57)
Local health network (%)	A	13.66	24.87	23.35	17.24	11.04	25.59	25.37	25.78	15.39	9.56
	B	16.53	11.13	14.20	10.79	5.79	0.60	1.33	6.12	13.61	25.01
	C	29.10	20.64	23.03	25.20	20.79	31.22	16.40	23.24	25.50	23.61
	D	31.66	36.19	23.84	26.56	19.45	41.65	56.37	35.46	18.61	14.51
	E	9.04	7.17	15.58	20.22	42.93	0.94	0.53	9.40	26.89	27.31
Medical vulnerability (%)^d^	Vulnerable	31.70	29.81	31.76	31.58	32.43	29.90	29.93	30.12	32.10	32.81
	Non-vulnerable	68.30	70.19	68.24	68.42	67.57	70.10	70.07	69.88	67.90	67.19
Clinical priority (%)^e^	1 (most urgent)	1.77	1.53	2.19	2.10	2.45	1.47	1.25	1.54	2.56	2.20
	2	12.38	13.42	16.44	18.40	22.16	11.31	13.21	13.88	17.57	18.61
	3	13.08	12.61	13.36	12.09	11.22	11.89	11.00	12.65	12.98	13.15
	4	27.95	26.26	25.06	23.77	22.71	27.51	26.04	25.10	24.44	25.39
	5 (least urgent)	44.82	46.17	42.95	43.64	41.47	47.81	48.50	46.84	42.45	40.65
Mean number of health conditions (s.d.)^f^		0.41 (0.79)	0.38 (0.76)	0.42 (0.81)	0.42 (0.79)	0.46 (0.84)	0.38 (0.76)	0.38 (0.76)	0.39 (0.78)	0.43 (0.82)	0.44 (0.81)
Health conditions (%)	Mental health^g^	5.54	5.18	6.49	7.20	7.61	4.57	4.42	4.94	6.75	8.05
	Major depression	1.96	1.95	1.77	1.92	1.94	1.25	1.82	1.73	1.98	2.34
	Addiction	0.57	0.63	0.92	0.97	1.20	0.50	0.43	0.60	0.83	1.20
	Active cancer	4.37	3.74	3.81	3.70	3.59	3.70	3.66	3.89	4.10	3.96
	Intellectual disability	0.39	0.40	0.43	0.57	0.68	0.48	0.50	0.49	0.45	0.48
	Diabetes	4.72	5.11	5.98	5.38	6.20	5.29	4.98	5.07	5.49	5.57
	COPD	3.52	3.21	4.06	4.07	5.06	3.41	3.05	3.62	4.27	4.25

*Abbreviations*: *very advant* very advantaged, *advant* advantaged, *med* medium, *disad* disadvantaged, *very disad* very disadvantaged

^a^Area-based material deprivation

^b^Area-based social deprivation

^c^s.d.: standard deviation

^d^Medical vulnerability is determined by the presence of at least one of 19 medical vulnerability codes, as pre-determined by the RAMQ, or being older of 70 year old

^e^Clinical priority level as assigned by the CWLs’ nurses (ranging from Priority 1 – most urgent, to Priority 5 – least urgent)

^f^Number of medical vulnerability codes present

^g^Menthal health problems excluding major depression

### Likelihood of attachment to a GP

Results of the analysis for our first objective (i.e. to examine the association between deprivation and the likelihood of attachment to a GP) are presented in Table 3. Likelihood of attachment to a GP was significantly lower for materially very advantaged (OR 0.75, CI 0.70–0.81) and advantaged patients (OR 0.88, CI 0.82–0.95) compared to very disadvantaged patients. In contrast, for social deprivation, likelihood of attachment was higher for very advantaged (OR 1.23, CI 1.15–1.32), advantaged (OR 1.21, CI 1.13–1.30) and medium (OR 1.19, CI 1.11–1.27) compared to very disadvantaged.

**Table 3:** Factors associated with likelihood of attachment to a GP through centralized waiting list among all subjects (n = 74, 859; June 2013 to May 2015) (multiple logistic regression)

Variables in the model		Attachment to a GP		
		Adjusted OR^i^	95% CI	*P*-value
Material deprivation^a^	Very advantaged	0.75	0.70; 0.81	<.0001
	Advantaged	0.88	0.82; 0.95	0.0004
	Medium	0.99	0.92; 1.06	0.77
	Disadvantaged	0.99	0.91; 1.06	0.67
	Very disadvantaged (ref.)^b^			
Social deprivation^c^	Very advantaged	1.23	1.15; 1.32	<.0001
	Advantaged	1.21	1.13; 1.30	<.0001
	Medium	1.19	1.11; 1.27	<.0001
	Disadvantaged	1.04	0.99; 1.10	0.11
	Very disadvantaged (ref.)^b^			
Sex	Male	1.07	1.02; 1.11	0.0019
	Female (ref.)^b^			
Age		0.99	0.98; 0.99	<.0001
Local health network	A	0.12	0.11; 0.13	<.0001
	B	0.42	0.39; 0.45	<.0001
	C	0.04	0.04; 0.05	<.0001
	D	0.43	0.41; 0.46	<.0001
	E (ref.)^b^			
Medical vulnerability^d^	Vulnerable	2.56	2.26; 2.84	<.0001
	Non-vulnerable (ref.)^b^			
Clinical priority^e^	1 (most urgent)	85.88	70.32; 104.89	<.0001
	2	13.43	12.39; 14.56	<.0001
	3	6.82	6.22; 7.48	<.0001
	4	2.13	2.03; 2.25	<.0001
	5 (least urgent) (ref.)^b^			
Number of health conditions^f^		0.95	0.91; 1.00	0.07
Health conditions	Mental health^g^	0.42	0.38; 0.46	<.0001
	Major depression	0.85	0.73; 0.98	0.03
	Addiction	0.39	0.31; 0.49	<.0001
	Active cancer	1.54	1.36; 1.74	<.0001
	Intellectual disability	0.72	0.54; 0.96	0.03
	Diabetes	1.46	1.32; 1.62	<.0001
	COPD	1.00	0.89; 1.12	0.98
	Absence of condition (ref.)^b^			
Wait time (days)^h^		1.00	0.99;1.00	0.54

^a^Area-based material deprivation

^b^Ref.: reference category

^c^Area-based social deprivation

^d^Medical vulnerability is determined by the presence of at least one of 19 medical vulnerability codes, as pre-determined by the RAMQ, or being older of 70 year old

^e^Clinical priority level as assigned by the CWLs’ nurses (ranging from Priority 1 – most urgent, to Priority 5 – least urgent)

^f^Number of medical vulnerability codes present

^g^Mental health problems excluding major depression

^h^Wait time: For patients waiting for attachment – number of days between CWL registration and May31^st^, 2015; For patients attached to a GP – number of days between CWL registration and attachment

^i^Adjusted OR: Odds ratio adjusted for all other variables presented in the table

While our focus is on material and social deprivation, several results relating to our control variables are of interest. Males were slightly more likely to be attached and increase in age slightly decreased likelihood of attachment. Moreover, likelihood of attachment to a GP varied between local health networks.

Medically vulnerable patients were 2.56 times more likely to be attached to a GP (OR 2.56, CI 2.26–2.84). Likelihood of attachment increased with urgency of clinical priority, with Priority 1 (most urgent) patients being nearly 86 times more likely to be attached to a GP than Priority 5 patients. Patients with active cancer (OR 1.54, CI 1.36–1.74) and with diabetes (OR 1.46, CI 1.32–1.62) were more likely to be attached to a GP, while patients with mental health problems (OR 0.42, CI 0.38–0.46), major depression (OR 0.85, CI 0.73–0.98), addiction (OR 0.39, CI 0.31–0.49) or intellectual disability (OR 0.72, CI 0.54–0.96) were more likely to remain on the waiting list.

### Wait time for attachment to a GP

Table 4 presents the results for our second objective of assessing the association between deprivation and wait time for attachment to a GP. The multilevel linear regression model included fixed patients level variables (level-1). While no GP level variables (level 2) were included in the model, the intra-class correlation suggests that 18% of the variance in wait times is explained by the GP to whom patients were attached. With regards to material deprivation, we observed a gradient in coefficients. For example, patients from very advantaged areas waiting 34 days less than patients from very disadvantaged areas (*p* < 0.001). Wait times for patients from medium socially disadvantaged areas waited about 15 days less than those for very socially disadvantaged areas (*p* = 0.0061). Wait times were not significantly different between patients from very socially disadvantaged areas and patients from other quintiles of deprivation.

**Table 4:** Factors associated with the wait time (days) for attachment to a GP among subjects who have been attached to a GP through centralized waiting list (*n* = 24, 958; June 2013 to May 2015) (Multilevel linear regression)

Variables in the model		Wait time for attachment to a GP		
		Coefficient Beta^h^	95% CI	P-value
Material deprivation^a^	Very advantaged	−34.20	−45.82; − 22.59	<.0001
	Advantaged	−24.92	−36.74; −13.10	<.0001
	Medium	−19.10	−30.67; − 7.54	0.0012
	Disadvantaged	−13.59	−24.70; − 2.48	0.0166
	Very disadvantaged (ref.)^b^			
Social deprivation^c^	Very advantaged	−7.14	−19.98; 5.69	0.2750
	Advantaged	−7.75	−20.82; 5.33	0.2455
	Medium	−15.92	−27.29; −4.55	0.0061
	Disadvantaged	−5.14	−13.91; 3.63	0.2505
	Very disadvantaged (ref.)^b^			
Sex	Female	−4.34	−11.14; 2.45	0.2098
	Male (ref.)^b^			
Age		1.30	1.10; 1.50	<.0001
Local health network	A	266.20	157.52; 374.89	0.0089
	B	7.68	−85.38; 100.73	0.7566
	C	182.57	88.95; 276.19	0.0139
	D	−41.65	− 121.13; 37.83	0.1528
	E (ref.)^b^			
Medical vulnerability^d^	Vulnerable	−73.72	− 59.46; − 87.99	<.0001
	Non-vulnerable (ref.)^b^			
Clinical priority^e^	1 (most urgent)	− 441.72	− 464.72; − 418.72	<.0001
	2	−296.38	− 308.66; − 284.10	<.0001
	3	− 246.75	−261.71; − 231.79	<.0001
	4	− 107.92	−118.98; −96.85	<.0001
	5 (least urgent) (ref.)^b^			
Number of health conditions^f^		−12.3450	−19.77; − 4.92	0.0011
Health conditions	Mental health^g^	78.50	63.00; 94.01	<.0001
	Major depression	21.01	−0.83; 42.84	0.06
	Addiction	122.07	84.46; 159.69	<.0001
	Active cancer	1.28	−14.79; 17.36	0.8754
	Intellectual disability	70.85	26.88; 114.82	0.0019
	Diabetes	−5.47	−19.98; 9.05	0.4591
	COPD	12.38	−4.94; 29.69	0.1604
	Absence of condition (ref.)^b^			

^a^Area-based material deprivation

^b^Ref.: reference category

^c^Area-based social deprivation

^d^Medical vulnerability is determined by the presence of at least one of 19 medical vulnerability codes, as pre-determined by the RAMQ, or being older of 70 year old

^e^Clinical priority level as assigned by the CWLs’ nurses (ranging from Priority 1 – most urgent, to Priority 5 – least urgent)

^f^Number of medical vulnerability codes present

^g^Mental health problems excluding major depression

^h^Beta adjusted for all other variables presented in the table and nested by GPs’ unique identifier

Age was also associated with a significant increase in wait time (β 1.30, CI 1.10–1.50). Wait time varied according to local health network, for instance patients from LHN A waited 266 days more than those from LHN E (*p* = 0.0089). With regards to morbidity variables, non-vulnerable patients (i.e. with no health conditions) waited 74 more days than medically vulnerable patients before being attached to a GP. Wait times decreased concurrently with increasing urgency of clinical priority level, with Priority 1 patients (most urgent) waiting 442 days less compared to Priority 5 (least urgent) patients. Also, for every additional health condition, patients waited 12 days less for attachment. Wait times were significantly longer for patients with a mental health problem (β 78.50, CI 63.00–94.01), addiction (β 122.07, CI 84.46–159.69) or intellectual disability (β 70.85, CI 26.88–114.82).

## Discussion

In sum, our results show that social and material deprivation are significantly associated with likelihood of attachment to a GP through CWLs and that material deprivation is significantly associated with wait time among patients attached to a GP though CWLs, independently of patients health needs and other control variables. It therefore seems that while CWLs may help reduce inequities in likelihood of attachment to a GP with regards to material deprivation, other inequities remain – our results highlighting a pro-socially advantaged gradient in likelihood of attachment to a GP and a pro-rich gradient in wait time for attachment to a GP. Our findings therefore suggest that CWLs may reduce some inequities, but that, despite a more standardized process for finding a GP, significant socio-economic inequities persist in attachment to a GP through CWLs. While our results do not provide information on the mechanisms leading to these inequities, we can hypothesize that these might be linked to patients’ abilities to access health care services, in this case the CWL and GP, as well as to the health care service’s characteristics, for instance the CWLs’ processes [60].

### CWLs may not be reaching patients from materially disadvantaged areas

The descriptive statistics of our population suggest that, compared to the general population of Quebec (expected 20% in each deprivation quintile), patients from very materially disadvantaged areas seem underrepresented on the CWLs, while patients from very socially disadvantaged areas seem to be overrepresented. Patients from more materially disadvantaged areas may have lower levels of health literacy [61–67]. This may make it more difficult for them to navigate the complex processes of the health care system [12, 68], such as registering on the CWL (e.g. knowing the CWL exists, finding local CWL contact information, filling out form). Moreover, the distribution of this study’s patients across deprivation indexes in the five LHNs selected might be different than the distribution observed at the provincial level (20% in each quintile). A previous report showed that two of the five LHNs were considered socially disadvantaged or socially and materially disadvantaged [69]. The other three LHNs were categorized as being socially and materially advantaged [69]. In order to account for this diversity, we controlled our analysis for the LHN. This may partially explain our finding that patients from more materially deprived areas seem underrepresented on CWLs for attachment to a GP, despite patients with lower income and education levels being more likely to not have a regular GP in Canada [6, 8–11].

Since the study, registration has been centralized provincially (single website) [70], simplifying the process somewhat for patients. However, it has been suggested that health systems should implement measures to better reach patients with low health literacy, not only by simplifying processes, but also by training health providers to better communicate with these patients and disseminating health information through community organisations, schools and the mass-media [71]. Therefore, promoting how to register on the CWL for attachment to a GP through various channels may help reach patients from materially disadvantaged areas.

### Patients from materially more advantaged areas and socially more disadvantaged areas less likely to be attached to a GP through CWLs

Our results also suggest that CWL patients from very materially advantaged and advantaged areas (richest) were significantly less likely to be attached to a GP through the CWLs compared to those from very materially disadvantaged areas (poorest) (OR 0.75, *p* < 0.0001 and OR 0.88, *p* = 0.0004), when controlling for health needs, age, sex, local health network and wait time. Conversely, we found that patients from socially very advantaged, advantaged and medium areas were more likely to be attached to a GP compared to patients from very disadvantaged areas (most isolated) (OR 1.23, 1.21 and 1.19 respectively, *p* < 0.0001). These results are opposite from the results presented in Table 2, suggesting that the control variables have an important influence on the association between social deprivation and likelihood of attachment to a GP, which is expected considering that medical vulnerability and clinical priorities, based on self-reported health conditions are used to assess patient health needs and urgency for attachment and financial incentives are based on patient health status. It is possible that patients from socially disadvantaged areas (more isolated) may be more likely to miss their first appointment with their new GP, which officialises the attachment, and are then sent back to the CWL. A recent report on CWL for attachment to a GP in Quebec found in 8.5% of cases where patients were not attached to the GP to whom they had been assigned, it was because they had missed their appointment [32]. Several studies have found that, in certain populations, patients with lower levels of social support were more likely to miss appointments [72–74]. Also, patients from more socially advantaged areas, by having someone such as a spouse to help explain their health status and advocate for their needs to the CWL nurse, may be more likely to be attached to a GP. However, the literature on the link between social deprivation and access to a GP is virtually inexistent. Our study contributes to this body of knowledge, but the mechanisms by which inequities would occur in likelihood, but not for wait time for attachment to a GP through CWLs remain unclear. Further research is needed to explain these results.

### Pro-materially advantaged gradient in wait time for attachment through CWLs

In terms of wait times for patients who had been attached to a GP through the CWLs, our findings show a significant pro-rich gradient in wait times for attachment, with patients from very materially advantaged areas (richest) having waited an average 34 days less than patients from very disadvantaged areas (poorest) (*p* < 0.0001). For social deprivation, our results show no significant differences other that patients from medium areas waited 15 days less for attachment than those from very disadvantaged areas (*p* = 0.0061). Patients are prioritized by the CWL nurse based on self-reported health information from their registration form and phone evaluation and then selected by the nurse for attachment as GPs become available. Therefore, it may be patients, as previously mentioned, that patients with higher health literacy and communication skills [63–66, 68], often those with higher education, income and from more materially advantaged areas [61, 62], are able to report their medical conditions and advocate for their need to be attached more clearly to the nurse which might explain shorter waiting times for attachment. These findings of a pro-rich gradient in wait times aligns with recent literature review by Siciliani which found a few studies that show pro-rich socio-economic inequities in wait times for access for other healthcare services such as elective procedures in publically funded health systems, despite efforts to equalize wait times [75].

### Different influences of deprivation on likelihood and wait times

Moreover, our results highlight that patients from materially disadvantaged areas were more likely to have been attached, but among those who had been attached, they had waited longer. One possible explanation for these seemingly contradictory results is that patients from areas with higher incomes, education levels and employment rates may have stronger abilities to advocate for themselves [63–66]. For instance, they may continue to look for a GP on their own (e.g. asking GPs they see at a walk-in clinic, having family members ask their GPs if they are attaching new patients) and find attachment outside the CWL, in which case the attachment would not appear in the SIGACO database. They would therefore appear to be less likely to be attached to a GP through the CWL when looking at both patients who are still waiting for attachment and those who have been attached. In addition, these seemingly contradictory results in likelihood and wait time may be due to significant differences between patients attached to a GP through CWLs (included in the analysis on wait time) and patients who remained on the CWLs (included in the analysis on likelihood, but not wait time), as suggested by the results in Table 1, which could have influenced the direction of the associations. Another variable that may influence likelihood of attachment is geographic distribution of GPs. However, very little research has been done to analyze the association between area deprivation and supply of GPs. Future research on this topic is warranted.

### Inequities in likelihood and wait time for attachment through CWLs for patients with mental health problems, addiction and intellectual disability

While the focus of our analysis was deprivation, our results also highlight that patients with mental health problems, major depression, addiction and intellectual disability were significantly less likely to be attached to a GP (respectively OR 0.42, *p* < 0.0001; OR 0.85, *p* = 0.03; OR 0.39, *p* < 0.0001; OR 0.72, *p* = 0.03) and patients with mental health problems, addiction and intellectual disability waited significantly longer for attachment to a GP (respectively 78.50 days, *p* < 0.0001; 122.07 days, *p* < 0.0001; 70.85 days, *p* = 0.0019) compared to patients without these conditions, when controlling for deprivation, clinical priority level and other variables. This suggests that these patients with complex psychosocial needs face certain inequities in attachment to a GP through CWLs. It seems essential that qualitative research be done to better understand the barriers these patients face, particularly as some of these conditions seem more prevalent in patients from high deprivation areas (Table 2).

### Including socio-economic status as a prioritization criteria

Our findings suggest that CWLs have not been entirely successful at reducing socio-economic inequities by prioritizing attachment to a GP based on patients’ medical needs, as we found significant differences in likelihood of being attached between quintiles of both social and material deprivation and wait time for attachment was significantly influenced by material deprivation. In a study by Noseworthy et al., it was reported that stakeholders (public and health administrators) felt that socio-economic status should not be used to prioritize patients for access to healthcare services because, if the guidelines were applied rigorously, patients’ needs would adequately be captured by medical criteria [22]. Therefore, one avenue to reduce inequities in attachment to a GP through CWLs may be to improve the respect of prioritization guidelines.

In Aidem’s study, while some stakeholders (public, practitioners, administrators, policy-makers) felt that while socio-economic status should not be included as a criterion, most agreed that conditions that disproportionately affect socio-economically disadvantaged groups such as mental health problems and addiction should be prioritized [76]. While this may be a solution to consider for policy, our results show that patients with mental health problems and addiction are less likely to be attached to a GP and wait longer for attachment even when controlling for area material and social deprivation, and therefore prioritizing these conditions in CWLs may not be sufficient to reduce inequities in attachment to a GP.

In Quebec, recent changes in provincial policies of family medicine groups now weigh patients from high social and material deprivation (i.e. poorest, most isolated) areas more heavily for resource allocations. Therefore, including socio-economic factors as criteria to prioritize patients in CWLs, albeit with less weight than medical criteria, may be acceptable to stakeholders and may be a potential solution to improving equity in attachment to a GP. An evaluation of this change in policy should be conducted to examine whether it acts as an incentive for GPs to attach patients from more disadvantaged areas.

### Study strengths and limitations

Our analysis has several limitations. First, the deprivation index is based on census data at the neighbourhood level and is used as a proxy measure for socio-economic status of individual patients [11, 41, 49, 50]. It is therefore possible for patients to live in a materially or socially disadvantaged area, but to have a higher socio-economic status or to have strong social support. In addition, as reported by Pampalon et al., in less population areas, social health inequalities could be underestimated using this index [41, 49]. In our study, one local health network (D) was located in a semi-urban area which may have affected our results and led to an underestimation of deprivation. However, by controlling for local health network, we minimized this potential bias. Furthermore, Pampalon’s social and material deprivation indexes do not include other patient characteristics such as immigration status or ethnicity which have been found to influence access to PHC in other studies [77–83]. While there are limitations to using the deprivation index, socio-economic variables (e.g. income, education, employment status, marital status) are often unavailable in administrative databases used by policy-makers. Second, our analysis focused on attachment to a GP through CWLs. However, formal attachment to a GP does not guarantee access to this GP and may therefore not fully reflect access to PHC. Continued access to the GP after attachment was not captured in the administrative database used, but warrants further research. Future research should evaluate the impact of attachment through these CWLs using longitudinal data. In addition, the administrative database used does not provide information on attachment to a GP through means other than the CWL (e.g. by asking a GP at a walk-in clinic). Patients registered on the CWLs may have found attachment to a GP elsewhere, but remain on the waiting list. Finally, many factors may influence attachment to a GP through CWLs such as geographic distribution and availability of primary care workforce and prioritization processes, etc. These factors, not captured by our variables, may interact with social and material deprivation and influence likelihood and wait time for attachment to a GP.

## Conclusions

Our findings suggest that centralized waiting lists have not been successful at standardizing the process of attachment to a GP sufficiently to eliminate socio-economic inequities. Policy makers should take these findings into consideration to adjust CWL processes. Further research on the potential mechanisms explaining these inequities, such as availability of GPs in disadvantaged areas, physicians’ willingness to attach disadvantaged patients and barriers disadvantaged patients may face in using CWLs is necessary to inform the implementation of potential solutions.

## Abbreviations

CI: Confidence interval
COPD: Chronic obstructive pulmonary disease
CWL: Centralized waiting list
GP: General Practitioner
LHN: Local health network
OECD: Organization for Economic Co-Operation and Development
OR: Odds ratio
PHC: Primary health care
RAMQ: *Régie de l’assurance maladie du Québec (*Quebec’s Health Insurance Board)
S.D.: Standard deviation
SIGACO: *Système d’information des guichets d’accès pour la clientele orpheline* (Quebec’s information system for centralized waiting lists for unattached patients)
